# The relationship between job satisfaction and turnover intention among nurses in Axum comprehensive and specialized hospital Tigray, Ethiopia

**DOI:** 10.1186/s12912-020-00468-0

**Published:** 2020-08-18

**Authors:** Dawit Gebregziabher, Eskedar Berhanie, Hagos Berihu, Addis Belstie, Girmay Teklay

**Affiliations:** Department of Nursing, College of Health Sciences and Comprehensive Specialized Referral Hospital, Aksum University, Aksum, Tigray Ethiopia

**Keywords:** Turnover intention, Intention to leave, Nurse

## Abstract

**Background:**

Nurses’ turnover is a global concern which if not handled well can harm the productivity of an organization. The high turnover rate of health workers critically affects the health system, particularly in countries with limited resources. Hence, effective retention strategies require clear identification of the variables at the workplace that determines nurses’ decision in staying in or leaving their employer organization. The aim of this study is to assess the relationship between job satisfaction and turnover intention among nurses in Axum Comprehensive and Specialized Hospital Tigray, Ethiopia.

**Methods:**

The research was conducted using institution based cross-sectional study design. A total of 148 nurses were included in the study using a systematic random sampling technique. The study was conducted from January 2018 to June, 2019. Data were collected using semi-structured self-administered questionnaires. It was entered into Epidemiological information version 7 and then analyzed using Statistical Package for Social Sciences version 22. Bivariate logistic regression analysis was employed to examine the statistical association between the outcome variable and selected independent variables. All variables with *P* value < 0.05 in the bivariate analysis were included in the multivariable analysis.

**Result:**

Out of 148 nurses, more than half (64.9%) had the intention to leave the organization. The finding of this study showed that the level of job satisfaction was significantly associated with the overall intention. Nurses who were unsatisfied on their job autonomy were 2.55 (95% CI: 1.194, 5.466) more likely to intend to leave their workplace than nurses who reported to be satisfied. Nurses who were unsatisfied on training opportunity were also 2.55 (95% CI: 1.167, 5.571) times more likely to leave their job than nurses who reported to be satisfied.

**Conclusion:**

The overall turnover intention of nurses was found to be high and significantly associated with dissatisfaction on autonomy, and training opportunity. Therefore, continuous effort should be made by ward managers to enhance nurses’ satisfaction onjob autonomy, and training opportunity.

## Background

Health care system is made up of different workforce. Among those nurses are the largest health professionals’ group within the healthcare workforce and majority of patients ‘related responsibilities lie under nurse professionals [1]. Nursing have a key role in health care systems especially in primary health care services where the nurse, as a member of a health care team, provides single-handed services as well as community-based integrated health care services in a safe, professional and ethical manner [2]. Hence, shortage of nursing professionals has implications for costs and efficiency, impacting the quality of patient care [3].

One of the several challenges in the history of the nursing profession is nurse turnover [4]. Nurses’ turnover refers to proportion of nurses that had to be replaced in a given period of time to average number of nurses [5] and generally viewed as the movement of nurses out of an organization [6].

Turnover can be either voluntary which is when an individual quits their job at their own request or it can be involuntary which is defined as the company initiating the turnover [4]. Turnover is preceded by turnover intention [7]. Intention to leave refers to conscious and deliberate willfulness to leave the organization and the turnover intention has been acknowledged as an excellent indicator of actual turnover [9]. As the turnover intention increases, actual turnover is also expected to increase [8].

Evidence suggests that high turnover of nurses leads to several negative impacts, such as decreased customer service levels, low patient safety, poor quality of nursing care and poor health care coordination which can lead to adverse patient outcomes. The residual staffs can be also affected by longer working shifts, heavier work load and job stress which leads to overtiredness and further turnover. Nurse turnover can also adversely impact the economy of health care organizations through increased separation and recruitment costs [3, 9, 10].

Turnover is a major problem worldwide especially in developing countries, particularly in Africa [11]***.*** In Ethiopia, despite having the highest number of health workers from sub-Saharan Africa, it has been suffering from shortage of health professionals and more than 50.2% the nurses intended to leave their job [12, 36]. According to WHO, the Africa health workforce observatory, the national estimated density of health workers, and the distribution of nurses was 0.84 per 1000 population and 0.03 per 1000 population, respectively. This showed the lowest threshold of worker required to achieve the set health system goals. Among the health workers, nurses were the second most unsatisfied next to physicians [13].

Nurses’ turnover rate is among the highest rates for health professional groups. Many studies showed that the decision of nurses to stay in or leave their workplace can be influenced by several factors including organization-related factors (such as hospital profitability, organizational working environment, and interpersonal relations), occupational or professional factors (include role- dilemmas, patient-initiated violence, work hours, risky working condition), employee factors (including age, educational preparation, and attitude towards the job), and exterior factors [9, 15].

According to the turnover theory by Mobley (1977), turnover intention starts withthe dissatisfaction among workforces, which forces them to search for substitute works. Hayes (2010) also concluded that, work satisfaction of nurses can be affected by several contributing factors such as nurses intra-personal, interpersonal, and extra-personal relationships [9, 16].

Previous studies have identified an array of factors associated with nurse turnover intention. But, most of them have focused on work-related factors such as high job demand, perceived autonomy at work, support from superiors or peers, and job satisfaction [17]. In addition, the dynamic modeling of turnover processes with the consideration of time or changes in job satisfaction [18]. Job satisfaction of early-career employees is defined as the psychological and physiological aspects of employees’ subjective responses to the working environment [19].

Job satisfaction is one of the more skilled turnover factors. It is an important area ofinvestigation because it is an antecedent that is associated with higher job performance, work ethics, higher worker enthusiasm, and less absenteeism, fatigue, and turnover [20].

The turnover rate could be reduced by investigating factors affecting nurses’ intention to leave [21]. Identification of these factors might lead to recommendations that could enable health institutions in Aksum town to retain more nurses and save costs on recruitment, selection, in-service education, and placement of nurses. The reduction in turnover intention helps to improve the quality of care rendered to the client. Different retention strategies like business process re-engineering, health care financing reforms, transfer policy, and performance-based motivation were implemented by the Tigray regional health bureau to reduce the problem [22]. Despite the implementation of the measures, the problem still exists.

Many researchers consider turnover as a problem and have attempted to answer “what really determines employee’s intention to leave” by investigating possible antecedents of employee’s intention to leave. Several pieces of literatures show results that are not consistent and conclusive about nurses ‟turnover intention. Furthermore, factors and retention strategies in one setting may not be applicable in other settings [23].

Internal motivation, emotional association of belongingness to the organization, and due regard for the organization’s goals is instrumental in fostering the construct of “turnover intention.” Therefore, despite these relationships, a relatively slight empirical study was conducted to investigate the association between job satisfaction and turnover, particularly from the African context. The rationale to conduct this study was that turnover intention is an emerging healthcare problem that is associated with costs and difficulties that Aksum University Comprehensive and Specialized Hospital is facing in the recruitment and retention of proficient nurses. Therefore, the identification of the factors associated with job leaving and the subsequent reduction in turnover intention may help improve the quality of care rendered to the client.

## Methods

Hospital-based cross-sectional study design was used to conduct the study in Axum University Comprehensive and Specialized Referral Hospital, one of the three-campuses found in Aksum University. The hospital was established four years back. It is located in northern Ethiopia, Central Zone of Tigray regional state and is around 1024 km from Addis Ababa, the capital city of Ethiopia. The hospital serves approximately 70,000–100,000 patients each year, but the exact number is not known. It has 300 beds with more than 10specialists and 140 medical doctors. There are about 200 residents and 50 interns, and 210 Nurses with different qualification. It was conducted from January 2018 –June 2019.

The sample size was calculated using single population proportion by considering the proportion of nurses’ turnover intention of 59.4% from previous study conducted in East Gojam [24]. At the 95% confidence level and a 5% margin of error, a total of 148 nurses was included using systematic random sampling.

The dependent variable was turnover intention. The independent variables were socio-demographic characteristics (sex, age, marital status, educational status, work experience, income level), context characteristics (work unit/department), and job satisfaction factors (autonomy, professional opportunities, scheduling, pay and benefits, support, relationship and interaction).

### Operational definitions

#### Turnover

The ratio of the number of nurses who have left during a given period divided by the average number of nurses in that organization during the same period [24].

#### Turnover intention

An individual’s perceived probability of permanently leaving the employing organization in the near future [24].

#### Nurse

Refers to anyone who had training in nursing profession at Diploma or higher level [24].

Data were collected using a structured self-administered questionnaire designed in English. The questionnaire was adapted [25, 27–31, 45] and modified from previous studies to suit the current research and arranged according to the particular objective. A single item scale of job satisfaction measure was used in this study as it is commonly accepted practice and has demonstrated high validity [33, 35, 38]. Measuring job satisfaction with a single item measure allows each study participants to rate their satisfaction based on job-related factors that are vital to them.

The turnover intention in this study refers to an individual’s perceived probability of permanently leaving the employing organization in the near future. The following three statements was used to measure nurses turnover intention: “I am actively searching for an alternative to this organization,” “As soon as it is possible, I will leave the organization” and “I often think about quitting my job”Each had three response options:(1) No; (2) Not sure; and (3) Yes. An average score of the three responses was used to measure the overall turnover intention. Hence, when study participants scored above the overall mean, they were considered as having the intention to leave the hospital, and those below the overall mean were categorized as not having the intention to leave the hospital [32]. In this study, internal consistency (Cronbach’s α) was tested and found to be 0. 83.

Job satisfaction survey, developed by Spector, was used based on nine job facets including pay, promotion, supervision, benefits, operating procedures of contingent rewards, co-workers, nature of work and communication [33]. The overall job satisfaction was rated on a single-item 5-point scale. Study participants were asked different questions related to job satisfaction factors (autonomy, professional opportunities, scheduling, support, relationships and interactions, as well as pay, and benefits). Each statement has five alternatives with a five-point scale (1 = Very dissatisfied, 2 = Dissatisfied, 3 = Neutral,4 = Satisfied, 5 = Very Satisfied) [34, 35, 38]. The level of reliability of this scale was acceptable with the Cronbach α of more than 0.86.

To ensure the validity of the scale used in this study, a pre-test was conducted in Shire General Hospital before the formal data collection. The pre-test included sixty-one nurses,29 males, and 32 females, selected from different service areas.

The data were entered using Epi info version and cleaned and analyzed using Statistical Package for Social Sciences (SPSS) version 22.0 software. Mean + SD, median ± interquartile range, and percentages was calculated for numerical data. Logistic regression was used to assess statistical association by calculating crude odds ratio and adjusted odds ratio to see the influence of independent variables on dependent variables. The significance of statistical association was tested using a 95% confidence interval and *P*-value < 0.05.

### Ethical consideration

Institution Review Board (IRB) of Aksum University, College of Health Sciences reviews the protocol to ensure full protection of the right of study subjects. Permission was given from Tigray regional health bureau to conduct the study. In addition, respondents were well informed about the purpose of the study. Data were collected after getting written informed consent from each participant using the consent form designed for this study. Data were treated confidentially and anonymously.

## Result

### Socio-demographic characteristics of participants

In this study, a total of 148 respondents participated with an overall response rate of 100%. More than one-third (39.9%) of the participants were females, and more than half (53.1%) were unmarried. The majority (71.6%) of the participantshad a bachelor degree in nursing. The mean (+ SD) age was 26.9(+ 3.244) years. Less than one-third of respondents were working in OPD (17.6%), in pediatrics (18.2%), in medical ward (29.1%), in OR (26.4%), and in surgical ward8.8%). More than half (52%) of the nurses have 1 up to 3 years of work experience and a similar percentage (52.7%) had no dependent family member(s) living with them. The mean (+ SD) monthly income of respondents was 4102.67 (±1263.69 SD) Ethiopian Birr **(**Table 1).

**Table 1:** Socio-demographic characteristics of nurses in Axum Comprehensive and specialized referral hospital Tigray, Ethiopia, 2018/2019(*n* = 148)

Variables	Category	Frequency (%)
Sex	Male	89 (60.1%)
	Female	59 (39.9%)
Age (years)	21–25	62 (41.9%)
	26–30	75 (50.7%)
	31–35	7 (4.7%)
	> 36	4 (2.7%)
Marital status	Married	65 (43.9%)
	Single	83 (56.1%)
Educational Status	Diploma	42 (28.4%)
	Degree	106 (71.6%)
Work Experience	< 1 year	20 (13.5%)
	1–3	77 (52.0%)
	3–5	29 (19.6%)
	> 5	22 (14.9%)
Work department	Medical Ward	43 (29.1%)
	Surgical ward	13 (8.8%)
	Pediatrics Ward	27 (18.2.%)
	OPD	26 (17.6%)
	OR	39 (26.4%)
Monthly income	< 3145	3 (2.0%)
	3145–3911	65 (43.9%)
	3912–4725	74 (50.0%)
	> 4725	6 (4.1%)
Had dependent family	Yes	70 (47.3%)
	No	78 (52.7%)

### Nurses turnover intention and job satisfaction

The overall turnover intention among the respondents was 64.9%. Turnover intention was high with a mean (+ SD) rating of 2.48(+.760) (on a scale of 1 to 3). Mean job satisfaction was 8.76(+ 1.79). Mean job satisfaction ratings for autonomy, pay and benefits, scheduling, and professional opportunities were 3.34, 3.32, 3.38 and 2.91, respectively (on a scale of 1 to 5).

The findings of the level of job satisfaction showed that the majority (52.7%) of the respondents were dissatisfied with autonomy. A similar level of dissatisfaction was found due to scheduling (57.4%) and limited professional opportunities (56.1%). More than one-third of the were not satisfied by pay and benefits (36.5%) and by the available support (34.5%) (Table 2).

**Table 2:** Level of Job satisfaction and organizational commitment by different dimensions among nurses in in Axum Comprehensive and specialized referral hospital Tigray, Ethiopia, Ethiopia, 2018/2019 (*n* = 148)

Variables	Category	Frequency	Percentage
The extent to make autonomous nursing care decision (Autonomy)	Satisfied	70	47.3%
	Dissatisfied	78	52.7%
Professional opportunities (Opportunities for further education)	Satisfied	65	43.9%
	Dissatisfied	83	56.1%
Scuduling (The time available to get through your work)	Satisfied	63	42.6%
	Dissatisfied	85	57.4%
The amount of support and guidance you receive from your supervisor (Support)	Satisfied	97	65.5%
	Dissatisfied	51	34.5%
Relationship and interaction (The relationship you have with other health care workers)	Satisfied	87	58.8%
	Dissatisfied	61	41.2%
The amount of payment you receive in comparisons with people in other occupation (Payment and benefits)	Satisfied	94	63.5%
	Dissatisfied	54	36.5%
The Match between your job description and what you do	Satisfied	84	56.8%
	Dissatisfied	64	43.2%
The extent to which you have adequate training for what you do	Satisfied	70	47.3%
	Dissatisfied	78	52.7%

### Factors associated with turnover intention among nurses

In bivariate analysis, autonomy, match between job description, training opportunity, guidance and support from supervisor, and payment and benefits showed significant statistical association with turnover intention.

On the other hand, a multivariable logistic regression analysis was done after adjusting for age, marital status, educational level, work department, work experience and monthly income. Only autonomy, and training opportunity were identified as significant predictors of nurses’ turnover intention. Nurses who were unsatisfied on their job autonomy were 2.55 (95% CI: 1.194,5.466) more likely to intend to leave their workplace than nurses who reported to be satisfied. Similarly, nurses who were unsatisfied on training opportunity were also 2.55 (95% CI: 1.167, 5.571) times more likely to leave their job than nurses who reported to be satisfied **(**Table 3).

**Table 3:** Bivariate and multivariable logistic regression analysis result for factors associated with turnover intention among nurses in Axum Comprehensive and specialized referral hospital Tigray, Ethiopia, 2018/2019(*n* = 148)

Variables	Category	Intention to leave		COR[95%CI]	AOR[95%CI]
		Yes	No		
The extent to make autonomous nursing care decision (Autonomy)	Satisfied	64 (66.7%)	19 (36.5%)	1	1
	Dissatisfied	32 (33.3%)	33 (63.5%)	1.470 (1.714,7.038) *	2.55 (1.194,5.466)^**^
Payment and benefits	Satisfied	68 (70.8%)	26 (50.0%)	1.00	1.00
	Dissatisfied	28 (29.2%)	26 (50.0%)	2.429 (1.207,4.888) ^*^	1.266 (0.565,2.838)
The Match between your job description and what you do	Satisfied	63 (65.6%)	21 (40.4%)	1.00	1.00
	Dissatisfied	33 (34.4%)	31 (59.6%)	2.818 (1.405,5.652) *	1.712 (0.782,3.750)
The extent to which you have adequate training for what you do	Satisfied	55 (57.3%)	15 (28.8%)	1.00	1.00
	Dissatisfied	41 (42.7%)	12 (71.2%)	3.309 (1.605,6.822) *	2.55 (1.167,5.571) ^**^
The amount of support and guidance you receive from your supervisor (Support)	Satisfied	69 (71.9%)	28 (53.8%)	1.00	
	Dissatisfied	27 (28.1%)	24 (46.2%)	2.190 (1.084,4.427) *	1.712 (0.782,3.750)

## Discussion

Given the increasing nurse turnover rates, it is vital to understand why nurses are leaving health care organizations or the profession altogether. In the present study the relationship between nurses’ job satisfaction and their turnover intention was examined.

This study revealed that the overall prevalence of turnover intention was 64.9%. This result washigher than findings of other similar studies conducted in Hong Kong (4.5%), South Uganda (26%), Islamic Azad University (Iran) (35%), Iran (32.7%), Saudi Arabia (40%), Japan (44.3%) and East Gojam (59.4%). But it was in line with a study conducted in Tabriz (Iran) (64%) and lower than the study conducted in Lebanon (67.5%) [14, 18, 20–24, 26, 37, 47]. This could be due to the differences in work load, infrastructure in the health institutions, work experience and environment.

Regarding job satisfaction, nurse’s turnover intention was significantly influenced by their satisfaction with their job. This supports the turnover theory which states that turnover intention begins with dissatisfaction among workers [16]. Of the job satisfaction factor, satisfaction with autonomy was significantly associated with nurse’s intention to leave. Nurses who were unsatisfied with autonomy were 2.55 times more likely to leave the hospital compared to those who weresatisfied (95% CI: 1.194, 5.466)]. This is in line with previous studies conducted in Tigray (Ethiopia) and Taiwan, Bangkok (Thailand) [25, 38, 40–44]. This could be due to the fact that nurses with higher autonomy in their working condition tend to have better critical-thinking skills and low psychological outlook to leave the organization. Therefore, having lower autonomy for their decisions might lead to low job satisfaction that contributes to their desire to stay in their organization.

Another significant predictor of turnover intention in the hospital was satisfaction with training opportunity. Nurses who were unsatisfied for their training opportunity in the organization were 2.55 times more likely to report they intended to leave the hospital (AOR = 2.55(95% CI, (1.167, 5.571)). This is consistent with previous studies conducted in Sidama zone (south Ethiopia), (East Gojam) (Northern Ethiopia), south Africa, Taiwan, Las Vegas (USA) which showed that job satisfaction has frequently been reported to be the most common predictor for nurses who decided to stay or leave, especially the training opportunity dimension [27, 30, 31, 43, 46]. The possible explanation for this could be career development and training opportunity in nursing promote job satisfaction, increased retention of nurses. Thus, nurses who perceive having low training opportunity may prefer to leave than stay.

However, previous studies in Tigray (Ethiopia), Bangkok (Thailand), Las Vegas (USA) have also shown that motivational factors like satisfaction with scheduling in addition to these autonomies and pay and benefits are important factors affecting turnover [25, 38, 45]. This is in contrary to our study which could probably be due to the differences between Ethiopian nurses and nurses from other countries in terms of medical culture, which are unique to each country.

### Limitation of the study

This study was conducted at one comprehensive referral hospital in Aksum city, Ethiopia. Therefore, the finding should be taken with caution since the participants were hospital nurses from a particular region of Ethiopia and do not represent all hospital employees in this country. Although relationships can be explored, since the study was cross sectional nature, it is not possible to infer causal directions between different job satisfaction variables. In addition, using single item scales to express the construct could make it difficult for this study to adequately address psychometric dimensions.

### Implications for practice

The results of this study underlined the importance for the hospital managers and human resource practitioners to develop multiple interventions at individual, leadership, and organizational level to increase job satisfaction and reduce turnover intention (including: designing orientation and mentorship programs for new nurse graduates, providing management training in transformational or relational leadership behavior and supportive supervision and designing team oriented interventions) [30, 39].

Furthermore, the finding this study will also provide nurse managers with importance enhancing the motivational quality of job characteristics and creating a healthy work environment, especially in terms of autonomy, career development and creating training opportunity. As autonomy is a threshold issue in nursing practice. Nursing directors should implement regular systematic clinical supervision to relieve the nurses emotional strain stemming from their work, as this intervention showed significant improvements in nurses’ autonomy. In addition, an increase in autonomy can be achieved by changing routines/responsibilities [48].

## Conclusion

The findings of this study revealed that Magnitude of nurses "turnover intention" in Axum Comprehensive and specialized referral hospital was found to be high and nurses who were unsatisfied with autonomy, and training opportunity, were more likely to leave the hospital than who were satisfied. Therefore, the hospital managers and human resource practitioners need to develop relevant programs which could increase nurses job satisfaction with regard to their job autonomy, training opportunityand there by foster the sense of responsibility and meaningfulness of nurses.

## Data Availability

The datasets used and/or analyzed during the current study available from the corresponding author on reasonable request.

## Abbreviations

CI: Confidence interval
AOR: Adjusted Odd Ratio
SPSS: Statistical Package for Social Sciences
AKUCSRH: Aksum University Comprehensive and Specialized Referral Hospital
OR: Operation Room
OPD: Out Patient Department
